# Cardiovascular magnetic resonance characterization of peri-infarct zone remodeling following myocardial infarction

**DOI:** 10.1186/1532-429X-14-24

**Published:** 2012-04-17

**Authors:** Karl H Schuleri, Marco Centola, Kristine S Evers, Adam Zviman, Robert Evers, João AC Lima, Albert C Lardo

**Affiliations:** 1 Johns Hopkins School of Medicine, Division of Cardiology, 1042 Ross Building, Baltimore, MD 21205, USA; 2 Azienda Ospedaliera San Paolo, Polo Universitario, Milan, Italy; 3 Radiology and Imaging Sciences, National Institutes of Health (NIH), Bethesda, MD, USA; 4 Department of Biomedical Engineering, Johns Hopkins University, Baltimore, MD, USA

**Keywords:** Cardiovascular magnetic resonance imaging, Myocardial infarction, Late gadolinium enhancement, Peri-infarct zone, Myocardial strain

## Abstract

**Background:**

Clinical studies implementing late gadolinium-enhanced (LGE) cardiovascular magnetic resonance (CMR) studies suggest that the peri-infarct zone (PIZ) contains a mixture of viable and non-viable myocytes, and is associated with greater susceptibility to ventricular tachycardia induction and adverse cardiac outcomes. However, CMR data assessing the temporal formation and functional remodeling characteristics of this complex region are limited. We intended to characterize early temporal changes in scar morphology and regional function in the PIZ.

**Methods and results:**

CMR studies were performed at six time points up to 90 days after induction of myocardial infarction (MI) in eight minipigs with reperfused, anterior-septal infarcts. Custom signal density threshold algorithms, based on the remote myocardium, were applied to define the infarct core and PIZ region for each time point. After the initial post-MI edema subsided, the PIZ decreased by 54% from day 10 to day 90 (*p *= 0.04). The size of infarct scar expanded by 14% and thinned by 56% from day 3 to 12 weeks (*p *= 0.004 and *p *< 0.001, respectively). LVEDV increased from 34.7. ± 2.2 ml to 47.8 ± 3.0 ml (day3 and week12, respectively; p < 0.001). At 30 days post-MI, regional circumferential strain was increased between the infarct scar and the PIZ (-2.1 ± 0.6 and -6.8 ± 0.9, respectively;* *p *< 0.05).

**Conclusions:**

The PIZ is dynamic and decreases in mass following reperfused MI. Tensile forces in the PIZ undergo changes following MI. Remodeling characteristics of the PIZ may provide mechanistic insights into the development of life-threatening arrhythmias and sudden cardiac death post-MI.

## Background

Despite substantial progress in risk stratification to identify susceptible patients and therapeutical advances, the risk of sudden death remains the highest in the first 30 days after myocardial infarction (MI) [1,2]. Estimates from recent clinical trials show an annualized sudden-death risk of 8% to 12% in the 3-month period after MI, even with optimal medical therapy [3]. Myocardial scar tissue is one of the most important structural substrates for sudden cardiac death. It has been demonstrated that islands of viable myocardium -- surrounded by regions of myocardial scar --can produce the substrate for monomorphic ventricular tachycardia (VT), which is a significant risk factor for sudden cardiac death [4].

Recent studies employing late gadolinium enhancement (LGE) CMR suggest that a mixture of viable and non-viable cells can be identified as the peri-infarct zone (PIZ), due to an intermediate CMR intensity level between normal (black) and infracted (white) myocardium. Although the detection of the PIZ is contributed to the partial volume effect in CMR acquisitions [5,6], this "grey zone" has been shown to be a predictor of post-MI mortality. Patients with a large PIZ volume are more susceptible to VT induction, and have worse cardiac outcomes [7,8].

However, PIZ data correlating tissue, substrate-specific characteristics of the temporal formation and functional remodeling of the PIZ, which occur concurrently with structural LV chamber remodeling, do not exist. The purpose of this study was to test the hypothesis that the peri-infarct zone undergoes spatial and functional changes over time in parallel with structural ventricular remodeling following reperfused MI.

## Methods

### Animal model

All animal studies were approved by the Johns Hopkins University Institutional Animal Care and Use Committee and comply with the "Guide for the Care and Use of Laboratory Animals" (NIH Publication no. 80-23, revised 1985). Fourteen female Göttingen minipigs, at 15 ± 1 months of age, were purchased from Marshall BioResources (North Rose, NY). Myocardial infarcts were created in a closed chest approach by 120 minutes balloon occlusion of the left anterior descending artery, followed by restoration of flow, as described in detail previously [9]. One animal did not survive the infarct procedure and 4 animals died during the follow-up period at day 3, day 4, day 30, and day 90. One Göttingen minipig could not be imaged at all time points because of poor venous accessibility.

### Cardiac magnetic resonance imaging protocols

CMR images were acquired sequentially -- prior to the infarction procedure -- at baseline (BSN) and at 3, 10, 30, 60, and 90 days post MI, using a 1.5 T MR scanner (CV/i, GE Medical Systems, Waukesha, WI).

Global LV function was assessed using a steady-state free precession pulse sequence with breath-holding acquisitions [10]. A total of six to eight contiguous short-axis slices were prescribed to cover the entire LV from base to apex. Image parameters were as follows: TR/TE = 4.2 ms /1.9 ms; flip angle = 45°; 256 × 160 matrix; 8 mm slice thickness/no gap; 125 kHZ; 28 cm FOV and 1 NSA.

To assess circumferential strain (Ecc), CMR tagged images were acquired with an electrocardiography-gated, segmented K-space, fast gradient- recalled- echo pulse sequence with spatial modulation of magnetization, to generate a grid tagged pattern [11,12]. Images were obtained at the same location as the cine-CMR images with image parameters as follows: TR/TE = 6.7 ms /3.2 ms; flip angle =12; 256 × 160 matrix, views/s: 4; 8-mm slice thickness/no gap; 31.25 kHz; 28 cm FOV; 1 NSA; and 6 pixels tagging space.

After an intravenous injection of Gd-DTPA (0.2 mmol/kg body weight, Magnevist, Berlex, Wayne, NJ), LGE CMR images were acquired 15 minutes later using an ECG-gated, breathhold, interleaved, inversion recovery, FGRE pulse sequence. LGE- CMR images were acquired in the same location as the short axis cine-images. Imaging parameters were TR/TE/TI = 7.3 ms, 3.3 ms and 180 to 240 ms; Flip angle = 25°; 256 × 196 matrix; 8 mm slice thickness/no gap; 31.2 kHZ; 28 cm field of view (FOV) and 2 NSA. Inversion recovery time was adjusted as needed to null the normal myocardium [13].

### CMR image analysis

CMR images, covering the entire ventricle, were analyzed using a custom research software package (Cine Tool, GE Medical Systems, Waukesha, WI). Infarct size and infarct mass in the LGE- CMR images were defined based on 3 standard deviations of the signal intensity from the remote mean (non-infarct) myocardium for the core infarct, and 2 standard deviations of the signal intensity from the remote mean for the PIZ; the full-width half-max (FWHM) method was also applied, as described in detail previously [6,7,14]. Infarct remodeling parameters were evaluated on delayed contrast enhanced CMR images using commercially available imaging analysis software (Sigma Scan® Pro5, Systat Software Inc., San Jose, CA). Infarct expansion and thinning were defined as follows:

$$\begin{array}{c} \text{Expansion}\;\text{Index}=\frac{\text{endocardial}\;\text{length}\;\text{of}\;\text{the}\;\text{infarct}\;\text{scar}}{\text{endocardial}\;\text{length}\;\text{of}\;\text{the}\;\text{whole}\;\text{ventricle}}\\ \text{Thinning}\;\text{Ratio = }\frac{\text{Thickness}\;\text{of}\;\text{infarct}\;\text{zone}}{\text{Thickness}\;\text{of}\;\text{remote}\;\text{zone}} \end{array}$$

Cine CMR images were analyzed with QMass MR® (Medis medical imaging systems, Leiden, The Netherlands). To evaluate global left ventricular (LV) function and volumes, and LV wall motion and wall thickness, endo- and epicardial borders of the LV were defined in the end-diastolic and end-systolic frame in contiguous slices, and then LV parameters and volumes were calculated. We assessed contraction in the mid-wall layer of 24 circumferential locations of the LV myocardium. Tagged images were quantitatively analyzed with a custom software package (Diagnosoft HARP, Diagnosoft Inc., Palo Alto, CA), as described in detail previously [15]. The peak systolic circumferential strain (Ecc) was determined from the strain map of each point. Negative Ecc values represent myocardial contraction, whereas values of increasing strain (toward positive values) reflect worsening of cardiac contractile function in that region. Less negative Ecc values represent hypokinetic myocardium. A value of 0 represents akinetic non-contractile myocardium, whereas a positive value represents dyskinetic myocardial segments.

For follow-up analysis, short axis LGE CMR images were compared to regional strain values. Images were divided in to 24 segments for detailed analysis. Regional strain values were matched with the infarct, PIZ, and remote areas, defined by LGE CMR images. Mechanical segmental dysfunction was then assessed by the circumferential strain uniformity ratio estimate (CURE) measurement, as previously described [16,17]. Briefly, circumferential strain (y-axis) was plotted versus sector position for the 24 evenly distributed segments in each slice (x-axis), and subjected to Fourier analysis. A perfectly synchronous ventricle provides a CURE value of 1, whereas for a perfectly dyssynchronous heart the CURE index is 0. Although the CURE index has been used previously to quantify dyssynchrony in the whole ventricle, we applied this method to index segmental dysfunction.

### Statistical analysis

All data are presented as mean ± standard error of the mean. Our data sets show Gaussian distribution. Follow-up data were analyzed using repeated measures of one way analysis of variance (ANOVA). Tukey's post hoc analysis was performed to compare variables at different time points. Strain values of infarct and PIZ were evaluated with a paired Student's t-test; *P*-values <0.05 were considered significant. All analyses were performed with commercially available software (GraphPad Software, LaJolla, CA)

## Results

We studied 8 minipigs sequentially with CMR and established that all enrolled animals followed the natural history of cardiac remodeling, after the index event, as described previously [18]. Changes in LVEF, LV volumes, and LV-mass are summarized in Table 1.

**Table 1:** Animal characteristic and left ventricular performance

	BSN	Day 3	Day 10	Day 30	Day 60	Day 90	*p*-value
**LVEF (%)**	52.7 ± 1.3	47.0 ± 2.4	43.3 ± 2.2	41.2 ± 2.0	38.1 ± 2.9	39.1 ± 2.8	**<0.001**

**LVSV (ml)**	21.0 ± 0.9	16.0 ± 0.8	16.8 ± 0.8	16.3 ± 1.1	17.6 ± 2.0	19.2 ± 1.9	**0.03**

**LVEDV (ml)**	40.3 ± 2.5	36.0 ± 2.5	39.2 ± 1.9	39.8 ± 2.6	46.3 ± 3.6	49.4 ± 3.5	**0.002**

**LVESV (ml)**	19.3 ± 1.7	19.8 ± 2.1	22.4 ± 1.7	23.5 ± 2.0	28.7 ± 2.7	30.2 ± 2.7	**<0.001**

**LVED mass (gram)**	32.1 ± 1.5	34.7 ± 1.7	34.4 ± 2.4	32.3 ± 1.6	35.8 ± 1.6	38.6 ± 1.8	**0.01**

**BW (kg)**	30.8 ± 1.5	29.7 ± 1.4	32.0 ± 1.8	31.7 ± 1.0	33.9 ± 1.2	35.1 ± 1.7	**0.1**

*LVEF *left ventricular ejection fraction, *LVSV *left ventricular systolic volume, *LVEDV *left

ventricular end-diastolic volume, *LVESV *left ventricular end-systolic volume, *BW *body

weight *BSN *baseline (CMR prior to MI)

* *p *< 0.05 versus BSN

^† ^*p *< 0.01 versus BSN

^‡ ^*p *< 0.01 versus Day 3

^§ ^*p *< 0.05 versus Day 3

^|| ^*p *< 0.05 versus Day 10

^¶ ^*p *< 0.05 versus Day 30

### Temporal changes of the PIZ

**Figure 1:**
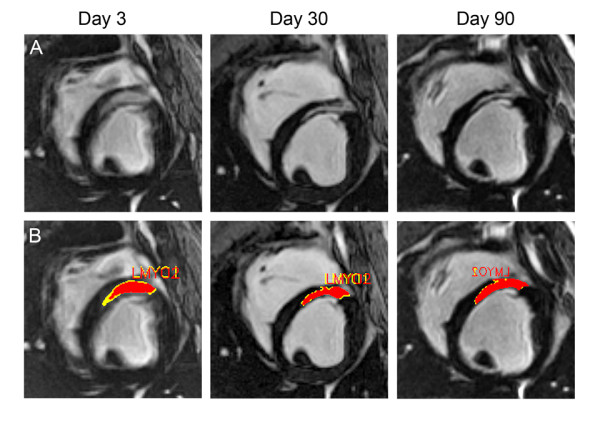
**Remodeling of the peri-infarct zone (PIZ) assessed by LGE-CMR**. (A) Example of one myocardial short axis slice followed over time until day 90 post-MI.(B) The same myocardial slices with computer –generated mask depicting the core infarct (red) and PIZ (yellow). The PIZ is most pronounced on day 3 and diminishes thereafter. After day 30 only little change in extent of the PIZ can be observed with CMR imaging.

**Figure 2:**
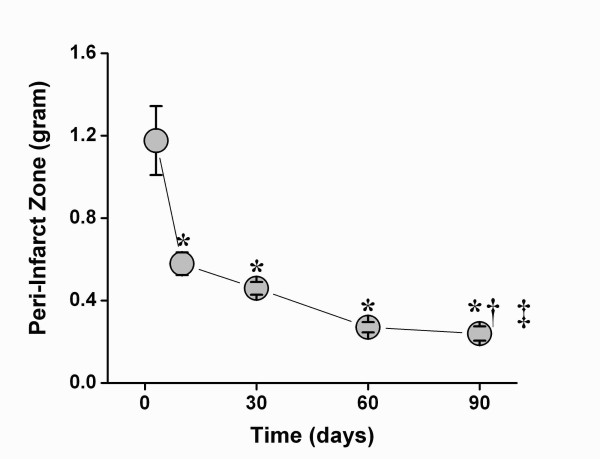
**PIZ remodeling over 90 days assessed by LGE-CMR imaging**. (A) The PIZ mass decreased consecutively until 90 days post-MI. The most pronounced drop was observed from day 3 to day 10. PIZ- mass decreased gradually until day 60, and only showed a minimal changes between day 60 and 90(* *p *< 0.01 versus day 3 post MI; **^† ^***p *< 0.05 versus day 10 post MI; repeated measures of ANOVA **^‡ ^***p *< 0.001).

**Figure 3:**
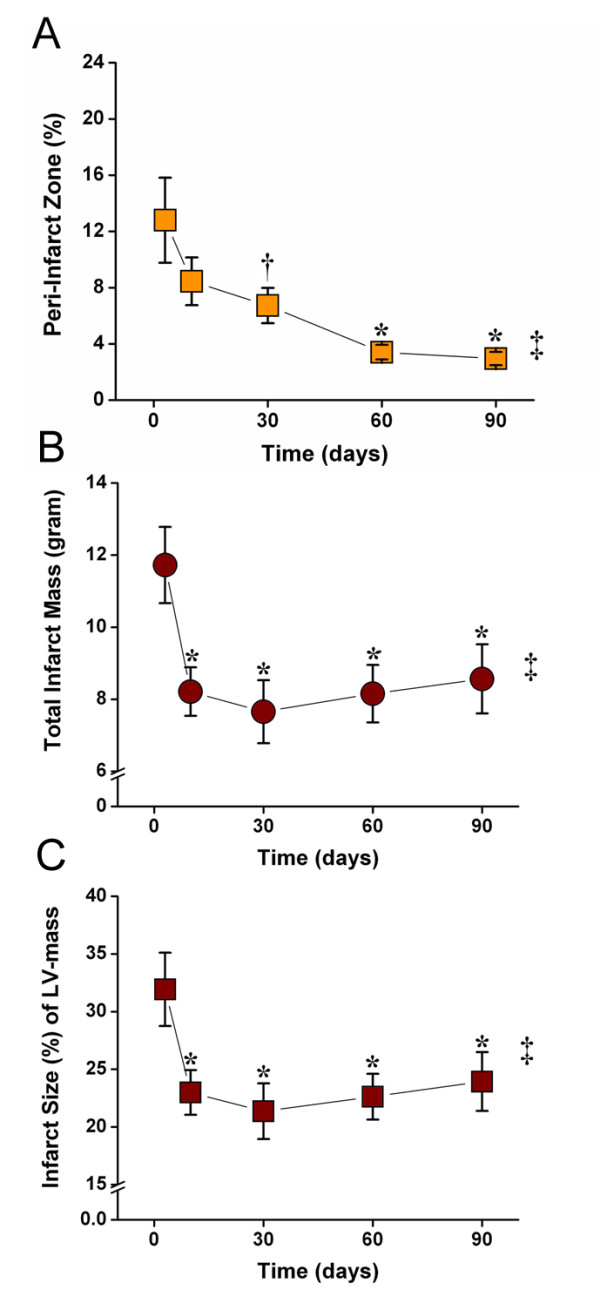
**LGE-CMR values of PIZ and infarct size remodeling**. PIZ expressed as percent of total infarct size decreased until day 60, and only showed a minimal changes between day 60 and 90(* *p *< 0.01 versus day 3 post MI; **^† ^***p *< 0.05 versus day 3 post MI; repeated measures of ANOVA **^‡ ^***p *< 0.001).(B) Absolute and (C) relative values of the infarct size show a continuous decline of infarct size until day 30. After 30 days the infarct mass and size increases until day 90 Consistent with infarct expansion (* p < 0.01 versus day 3 post MI; repeated measures of ANOVA **^‡ ^***p *< 0.001).

Three days post MI the infarct territory was large, accompanied by the largest peri-infarct zone detected by CMR (Figure 1). At 10 days, the infarct territory and the PIZ decreased by 28 ± 5% percent and 46 ± 6% percent, respectively -- using the 2 and 3 standard deviations threshold. We also applied the FWHM criterion and found a decrease from 5.9 ± 0.5 gram to 3.5 ± 0.4 gram at day 3 to day 10, respectively, (p < 0.001) -- which represent a 39.6% in PIZ. There were no differences in decrease with either method (p = 0.41). The myocardial scar expanded and thinned until day 90, consistent with ongoing remodeling processes (Table 2). The infarct scar mass decreased until week 4, and then modestly increased thereafter in accordance with infarct expansion. In contrast, the PIZ decreased continuously until day 90. The results of the absolute and derived values for the infarct size and the peri-infarct zone -- using the 2 and 3 standard deviation threshold --- are shown in Figures 2 and 3. The PIZ analyzed with the FWHM criterion followed the same pattern.

**Table 2:** Left ventricular remodeling indices

	Day 3	Day 10	Day 30	Day 60	Day 90	*p *- Value
Expansion Index	3.4 ± 0.29	3.21 ± 0.22	3.76 ± 0.31	3.72 ± 0.30	4.30 ± 0.47*^†^	0.01

Thinning Ratio	1.3 ± 0.10	1.19 ± 0.11	0.92 ± 0.05^‡§^	0.90 ± 0.04^‡§^	0.77 ± 0.06^‡†^	<0.01

* *p *< 0.05 versus Day 3

^† ^*p *< 0.01 versus Day 10

^‡ ^*p *< 0.01 versus Day 3

^§ ^*p *< 0.05 versus Day 10

### Regional parameters of remodeling

**Figure 4:**
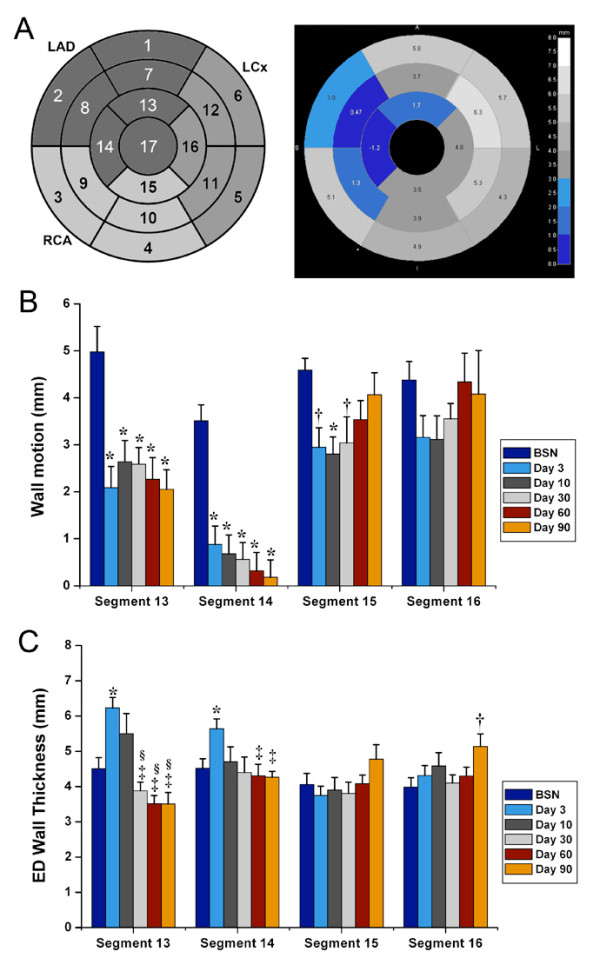
**Regional remodeling of the left ventricle after myocardial infarction**. (A) Wall motion (ES-ED wall thickness) and endiastolic (ED) wall thickness of the whole left ventricle expressed in 17-segment model in relation to coronary artery supply. The polar plot on the right is an example of wall motion at 90 days post-MI. The reduced wall motion in the apical and mid-wall region in the LAD territory is revealed by the different shades of blue. (B) Wall motion in the apical segments decreases continuously during the 90 day follow-up in the infracted LAD territory (segment 13 and 14; * *p *< 0.01 versus baseline [BSN]; CMR acquisitions prior to MI), while it only decreases initially in the non-infarct related arteries (segment 15; * *p *< 0.01 versus BSN, ^† ^*p *< 0.05 versus BSN) and recovers during the follow-up (*p *= NS versus BSN). (C) ED wall thickness increase with post-MI edema in segments 13 and 14 (* *p *< 0.01 versus BSN) and decreases after edema has been absorbed (^‡ ^*p *< 0.01 versus Day 3, ^§ ^*p *< 0.05 versus Day 10). ED wall thickness of segment 16 at day 90 indicates compensatory hypertrophy of the remote, non-infarcted territory, to compensate for the thinning infarct scar (^† ^p < 0.05 versus BSN).

**Figure 5:**
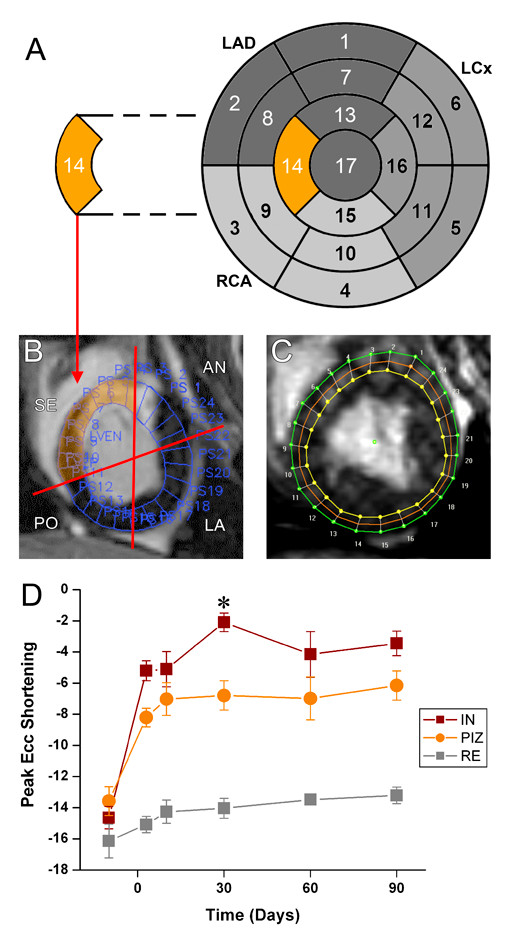
**Strain analysis in the peri-infarct zone in short axis slices**. (A) The 17-segment analysis provides an overview regional function in the whole left ventricle. (B) However, data from the infarct, the PIZ and non-infarcted areas have to be combined, and are therefore averaged. The septal (SE) area highlighted in orange represents segment 14. (SE = septal; AN = anterior; LA = lateral; PO = posterior). (C,D) To assess regional strain in the PIZ we sub-divided the short axis slices in 24 segments and matched each segment with the corresponding segment from the tagging analysis for Ecc and Err analysis, respectively.(D) At 30 days post-MI peak Ecc is different between the infarct scar tissue (IN) and the PIZ (* *p *< 0.05 versus IN). (F) At 3 days and 30 days post-MI the Err shows different strain values in the IN and PIZ(* *p *< 0.05 versus IN). (RE = remote moyocardium, PIZ = peri-infarct zone, IN = myocardial infarct scar).

We examined wall motion and end-diastolic wall thickness as regional remodeling parameters by using the American Heart Association 17-segment model. As shown in Figure 4, the end-diastolic wall thickness initially increased after acute MI, in the infarcted territories, and then decreased over time in parallel with a loss of regional function. We analyzed the course of myocardial function in the PIZ in detail by following single slices over time (Figure 4). Since the 17-segment model averages data from large sectors of the left ventricle, we focused on regional strain analysis in single slices divided by the 24 segments (Figure 5). At day 30, Ecc showed increased strain values in the PIZ, compared to the infarct scar (*p *< 0.05), which is consistent with a tethering effect (of the infarct scar on the viable myocytes) in the PIZ (Figure 5). The time to peak strain also showed a delay between the infarct scar and the PIZ (203.0 ± 11.9 ms vs.171.2 ± 7.4 ms, respectively; *p *< 0.05). In addition, segmental contractive dysfunction, quantified by CURE, was increased acutely after MI (BSN vs day3; *p *< 0.05) and showed the most dysfunction 30 days post MI, represented by the lowest CURE value (0.74 ± 0.06).

## Discussion

This is the first report to use CMR to characterize PIZ remodeling after myocardial infarction. There are two major findings in this study: 1) temporal changes in PIZ size can be detected with LGE-CMR, and 2) myocardial strain patterns change during the post-MI remodeling process.

### Post-MI remodeling and the underlying substrate for PIZ-imaging

Cardiac wound repair, after MI, involves temporarily overlapping phases which include an inflammatory phase and tissue remodeling phase. The first phase starts shortly after coronary artery occlusion-- with or without reperfusion-- and involves degradation of normal extracellular matrix, invasion of inflammatory cells at the site of initial injury, and the induction of bioactive peptides and cytokines [19]. Therefore, the early imaged MI substrate is characterized by a high cellularity, which is replaced by dense collagen fibers. These synthetic and degradative events, within the myocardial extra-cellular matrix, occur in a time and region-dependent manner following MI - which is reflected by histological and ultrastructural morphological changes. Within the MI region, the newly formed scar provides a means to tether viable myocyte fascicles, and thereby forms a substrate to resist deformation from the intra-cavitary stresses, generated during the cardiac cycle [20,21].

### LGE-CMR imaging and PIZ assessment

Early CMR acquisitions for infarct size quantification in the acute phase are complicated by post-MI edema, which leads to an overestimation of the exact MI-size. Although the assessment of the PIZ may be influenced by the tissue edema, the PIZ (by CMR) still reflects the mixture of viable and infarcted myocardium.

Different techniques for analysis have been previously used to determine the PIZ in CMR. Specifically, the assessment of the PIZ is either defined with threshold techniques using standard deviations to the remote myocardium [8], or based on modified FWHM criterion applying different signal intensities [7,22]. All of these methods claim to match the visual assessment of the PIZ by LGE-CMR. However, with the lack of quantitative histological reference, standard to index the heterogeneity of the PIZ, it is difficult to determine which method is superior over the other. In our analysis, we chose to use the 2 and 3 standard deviations threshold for this report and we found the best agreement with our visual assessment of the PIZ on LGE CMR images. In addition, we quantitatively assessed the PIZ with the FWHM criterion and found a similar remodeling pattern. We discovered that the quantified PIZ mass was larger with the FWHM method, compared to the threshold technique -- which confirms previous reports [7,8].

CMR has been used for risk stratification for ventricular arrhythmias following MI. Studies conducted in the 1980s show that a low LVEF predicts the risk of death after MI [23,24], and this data is currently used as one of the main criteria for ICD implantation [25]. Subsequent studies demonstrate that infarct surface area and mass-- measured by CMR-- can more accurately identify patients at risk and present a substrate for monomorphic VT, when compared to LVEF [26]. The ability to perform more detailed morphological analysis of the infarct scar will allow refined information on risk stratification [7,8].

Histologically, the early period of remodeling is characterized by the ongoing changes of the extracellular matrix and cell composition of tissue scar substrate, whereas the later period is characterized by a fixed substrate of cardiomyoctes and well-defined collagen scar [ref]. We demonstrate that the PIZ--assessed by LGE-CMR imaging-- undergoes significant changes early after MI as well. Until day 10, the PIZ decreases rapidly in mass and the PIZ mass stabilizes thereafter. Our data suggests that after 4 weeks post- MI, the PIZ can reliably evaluate structural remodeling. Additional information for regional strain analysis was acquired, which provides valuable information about the tensile forces working on the scar tissue and in the PIZ.

Recently, Fernandes *et al*. showed that enhanced PIZ function, defined as greater Ecc and earlier time to peak Ecc, was associated with inducibility in patients with post MI heart failure [27]. In our research, we did not see enhanced PIZ function at any particular time point during the remodeling process up to 90 days. However, at 30 days post-MI, we did observe a significant difference in Ecc and time to peak Ecc, between the infarct scar and PIZ, suggesting heightened mechanical stress working at the interface of viable and infarct tissue (Figure 6) [28,29]. Tensile forces working on the viable myocytes -- neighboring the scar tissue -- have been proposed for decades as crucial patho-physiological mechanisms, involved in post-MI remodeling and infarct expansion [18]. Until now, this concept could not be experimentally verified using non-invasive imaging methods. A recent study demonstrated that circumferential strain rates are independent predictors of outcomes after MI and are predictive of cardiac remodeling following MI [30]. However, Sasano *et al*. reported the highest inducibility in a porcine model, 30 days after MI-induction [31]. This is the same time-point that we observed the highest difference of strain between infracted area and PIZ in our pigs. The study of Sasano *et al*. and our findings suggest that the underlying pathophysiology (of post-MI VT) can be elucidated with imaging techniques.

### Limitations

The most significant limitation in this animal study was the inability to perform serial VT inducibilty studies with programmed stimulation. Having this data would help to determine the relationship between the imaged tissue substrate and susceptibility to VT. Another limitation was that all of the infarcts were created in the LAD territory, and were transmural. Strain patterns in the PIZ for non-transmural infarcts are expected to be very different from those in transmural infarcts, but our research did not explore this theory. Further, we did not acquire T2-weighted CMR images to quantify the post-MI edema, nor did we use any medications that are currently administered to patients in the post-MI period.

## Conclusions

The PIZ is a complex tissue substrate that changes its composition and decreases in mass, following reperfused MI. The difference in tensile strain forces, between the infarct scar and the PIZ, is reached approximately 30 days after MI. After this time, the underlying tissue substrate is mainly composed of viable myocytes and clear defined collagen tissue. Myocardial strain characteristics, combined with the PIZ for the assessment of LGE-CMR images, may provide important clinical information for the development of life-threatening arrhythmias, as well as sudden cardiac death post-MI.

## Abbreviations

BSN: Baseline; CMR: Cardiovascular magnetic resonance; CURE: Circumferential strain uniformity ratio estimate; Ecc: Circumferential strain; Err: Radial strain; FWHM: Full-width half max; LGE: Late gadolinium enhancement; LV: Left ventricle/ventricular; LVEDV: Left ventricular end-diastolic volume; LVESV: Left ventricular end-systolic volume; LVEF: Left ventricular ejection fraction; LVSV: Left ventricular systolic volume; MI: Myocardial infarction; PIZ: Peri-infarct zone; RE: Remote myocardium; VT: Ventricular tachycardia.

## Competing interests

The authors declare that they have no competing interests.

## Authors' contributions

KHS, planned, and performed experiments, supervised CMR acquisitions, analyzed data and images, and wrote the manuscript. MC planned, and performed experiments. KSE and AZ performed imaging analysis. RE acquired CMR data. JACL edited the manuscript. ACL supervised imaging analysis, and edited the manuscript. All authors read and approved the final manuscript.
